# Supplementary material for: Dysfunctional natural killer cells can be reprogrammed to regain anti-tumor activity
Source: EMBO J. 2024 Apr 18;43(13):2552–81. doi: 10.1038/s44318-024-00094-5 (PMC11217363; doi:10.1038/s44318-024-00094-5)
Supplement: Supplementary file 1 — Appendix [file 44318_2024_94_MOESM1_ESM.pdf]

Appendix

Dysfunctional natural killer cells can be reprogrammed to regain anti-tumor activity

Batel Sabag[1], Abhishek Puthenveetil[1], Moria Levy[1], Noah Joseph[1], Tirtza Doniger[1], Orly Yaron[1], Sarit Karako-Lampert[1], Itay Lazar[1], Fatima Awwad[1], Shahar Ashkenazi[1] and Mira Barda-Saad[1*]

Affiliations: [1]The Mina and Everard Goodman Faculty of Life Sciences, Bar-Ilan University, Ramat-Gan 5290002, Israel.

*To whom correspondence should be addressed: Prof. Mira Barda-Saad, The Mina and Everard Goodman Faculty of Life Sciences, Bar-Ilan University, Ramat-Gan 5290002, Israel. Tel.: +972-3-5317311; E-mail: Mira.Barda-Saad@biu.ac.il

Table of Contents:

1. Appendix Figure S1 ----- Page 1
2. Appendix Table S1 ----- Pages 2-8

Appendix Figure S1:

A For figure 2G

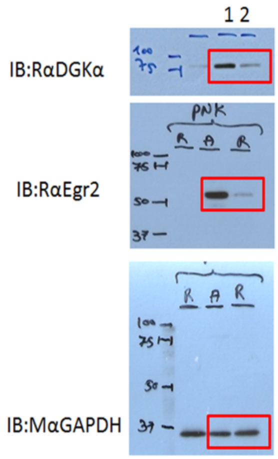

1 – Anergic NK cells
2 – Responsive NK cells

B For figure 4D

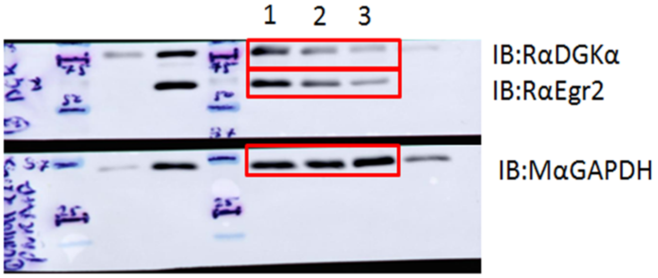

1 – Anergic NS
2 – Anergic siRNA Egr2
3 – Responsive NS

C For figure S4A

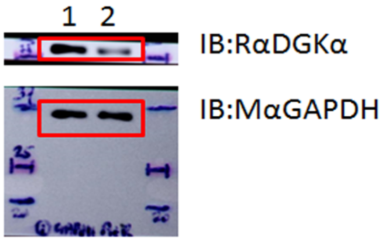

1 – Anergic NS
2 – Anergic siRNA DGKα

Appendix Figure S1 Legend: Representative raw uncut blots depicted in the manuscript.

Appendix Table S1:

Reagent type (species) or resource	Designation	Source or reference	Identifiers	Additional information
Antibody	EGR2	Sigma Aldrich	AV100880	IB: 1:1000 (7 µL)<br>IF: 1:100(1 µL)
Antibody	DGKα	Proteintech	11547-1-AP	IB: 1:1000 (7 µL)<br>IF: 1:100 (1 µL)
Antibody	DGKz	Abcam	Ab239081	IB: 1:1000 (7 µL)
Antibody	Anti-human GAPDH (0411) (mouse monoclonal)	Santa Cruz	SC-47724	IB: 1:1000 (7 µL)
Antibody	Anti-human pSHP-1 (S591) (rabbit polyclonal)	ECM Biosciences	Sp-1531	IB: 1:1000 (7 µL)<br>IF: 1:500 (0.6 µL)
Antibody	Anti-human pPLCγ1 (Y783) (rabbit polyclonal)	Cell Signaling	#2821	IB: 1:1000 (7 µL)<br>IF: 1:250 (0.6 µL)
Antibody	Anti-human pPLCγ2(Y1217) (rabbit polyclonal)	Cell Signaling	#3871	IF: 1:250 (0.6 µL)
Antibody	Anti-human pERK(Y204) Clone E-4	Santa Cruz	SC-7383	IF: 1:100 (1 µL)
Antibody	Anti-human CD16	Biolegend	302002	IF: 1:100 (1 µL)

Reagent type (species) or resource	Designation	Source or reference	Identifiers	Additional information
Antibody	Anti-human CD335 (NKp46) mouse monoclonal antibody<br>Clone 461-G1	MERCK	MABF2059	IF: 1:100 (1 µL)
Antibody	Anti-human CD226(DNAM-1) Clone D-11	Santa-Cruz	SC-376736	IF: 1:50 (1 µL)
Antibody	Anti-human CD244 (2B4) Clone HD2	Santa-Cruz	SC-53596	IF: 1:25 (1 µL)
Antibody	Anti-human CD160 APC	Biolegend	341203	IF: 1:100 (1 µL)
Antibody	Anti-human CD45 FITC	BD	555482	IF: 1: 100 (1 µL)
Antibody	Anti-human CD56 FITC	BD	562794	IF: 1: 100 (1 µL)
Antibody	Anti-human CD3 PE-Cy5	BD	555334	IF: 1: 100 (1 µL)
Antibody	Anti-human NFAT1(25A10.D 6.D2)	Abcam	Ab2722	IF: 1: 100 (1 µL)
Antibody	Anti-human NFAT2(7A6)	Abcam	Ab2796	IF: 1: 100 (1 µL)
Antibody	Goat anti-mouse HRP	Jackson Laboratory	#115-035-003	1:10,000 (1 µL)
Antibody	Goat anti-rabbit HRP	Santa Cruz	Sc-2004	1:10,000 (1 µL)

Reagent type (species) or resource	Designation	Source or reference	Identifiers	Additional information
Antibody	Anti-human KIR2D PE conjugated	Miltenyi Biotec	130-123-710	1:10 (10 µL)
Antibody	Anti-human CD158e1 (KIR3DL1, NKB1)-PE conjugated	BioLegend	#312708	1:10 (10 µL)
Antibody	Anti-human CD107a (LAMP-1) (mouse monoclonal)	BioLegend	#328602	1:20 (2.5 µL)
Antibody	Anti-human CD366 (Tim-3) APC	BioLegend	#345011	1:100 (1 µL)
Antibody	Anti-human TIGIT (VSTM3) APC	BioLegend	#372705	1:100 (1 µL)
Antibody	Anti-human CD279 (PD-1) APC	BioLegend	#329908	1:50 (1 µL)
Antibody	Alexa Fluor-conjugated 488 (goat polyclonal) anti-rabbit IgG (H+L) Highly Cross-Adsorbed Secondary Antibody	Invitrogen	A11034	IF: 1:2000 (1 µL)

Reagent type (species) or resource	Designation	Source or reference	Identifiers	Additional information
Antibody	Alexa Fluor-conjugated 488 goat polyclonal anti-mouse IgG (H+L)	Jackson Laboratory	115-545-146	IF: 1:2000 (1 µL)
Antibody	Alexa Fluor-conjugated 647goat polyclonal anti-Rabbit IgG (H+L)	Invitrogen	A11034	IF: 1:2000 (1 µL)
Sequence-based reagent	siRNA: target *DGKA* gen e	Sigma-Aldrich	SASI_Hs01_0 0072301	
Sequence-based reagent	siRNA: target *DGKZ* gene	Sigma-Aldrich	SASI_Hs02_0 0324291	
Sequence-based reagent	esiRNA: target *EGR2* gene	Sigma-Aldrich	EHU124311	
Sequence-based reagent	siRNA: nonspecific target	Sigma-Aldrich		5′ UAGCGACUAAACACAUCAA 3′,5′UAAGGCUAUGAAGAGAUAC3 ′, 5′AUGUAUUGGCCUGUAUUAG3′, 5′ AUGAACGUGAAUUGCUCAA 3′, and 5′ UGGUUUACAUGUCGACUAA3′
Chemical compound, drug	FGF	Sigma-Aldrich	SRP3043	0.1ug/mL

Reagent type (species) or resource	Designation	Source or reference	Identifiers	Additional information
Chemical compound, drug	TGF-Beta	Abcam	ab50036	20ng/mL
Chemical compound, drug	EGF	Sigma-Aldrich	SRP3027	0.18ug/mL
Chemical compound, drug	Indo-1-AM	BIOTIUM	#50044	
Chemical compound, drug	DGK inhibitor I	Sigma-Aldrich	D5919	
Chemical compound, drug	[^{35}S]Met	PerkinElmer	NEG009L005MC	
Chemical compound, drug	Monensin	BioLegend	#420701	
Chemical reagent	Mirus	Ingenio Solution	MIR50111	
Chemical compound, reagent	Enhanced chemiluminescence	PerkinElmer, Life Gene	NEL105001EA,AC2103	
Chemical compound, reagent	MATRIGEL (with Phenol red)	Corning	FAL356234	
Chemical compound,	MATRIGEL (w/o Phenol red)	Corning	FAL354234	

**Reagent type (species) or resource**	**Designation**	**Source or reference**	**Identifiers**	**Additional information**
reagent				
Commercial assay or kit	NK Cell negative selection enrichment kit	STEMCELL Technologies	Cat# 19055	
Commercial assay or kit	Quick-RNA isolation MicroPrep kit	Zymo research	R1050	
Software, algorithm	GraphPad Prism	V9.0.1	RRID:SCR_002798	
Software, algorithm	Adobe Photoshop	CC2019		
Software, algorithm	Biorender		RRID:SCR_018361	
Software, algorithm	FACS Diva			
Software, algorithm	ImageJ 1.53c	https://imagej.nih.gov/	RRID:SCR_003070	
Cell line (*H. sapiens*)	721.221		RRID:CVCL_6263;ATCC: CRL-1855	Kind gift from Prof. Ofer Mandelboim. STR profiling was done by ATCC
Cell line (*H. sapiens*)	721.221 HLA-Cw7		RRID:CVCL_6263;ATCC: CRL-1855	Kind gift from Prof. Ofer Mandelboim. STR profiling was done by ATCC
Cell line (*H. sapiens*)	K562		RRID:CVCL_0004;DSMZ no. ACC-434	Kind gift from Prof. Ofer Mandelboim. STR profiling testing was done by DSMZ
Cell line (*H. sapiens*)	PANC-1		RRID:CVCL_0480; ATCC:CRL14	

Reagent type (species) or resource	Designation	Source or reference	Identifiers	Additional information
			69	
Biological sample (*H. sapiens*)	PBMCs			Blood samples from healthy donors were provided by Magen David Adom (MDA; Israeli National Blood Bank)Donor's identification information remained anonymous
Strain, strain background (*Mus musculus*, female)	NOD-Rag1^{null}IL2rg^{null} (RAG)	Jackson Labs	NOD-Rag1^{null}IL2rg^{null} (RAG)	Details are listed in In vivo conditions and study design table

Appendix Table S1: Resources (antibodies, reagents or species) used in the manuscript.
