# Supplementary material for: Dysfunctional natural killer cells can be reprogrammed to regain anti-tumor activity
Source: EMBO J. 2024 Apr 18;43(13):2552–81. doi: 10.1038/s44318-024-00094-5 (PMC11217363; doi:10.1038/s44318-024-00094-5)
Supplement: Supplementary file 3 — Source data Fig. 2 [file 44318_2024_94_MOESM3_ESM.zip › Figure 2/Representative blot - Fig. 2I.pptx]

## Slide 1
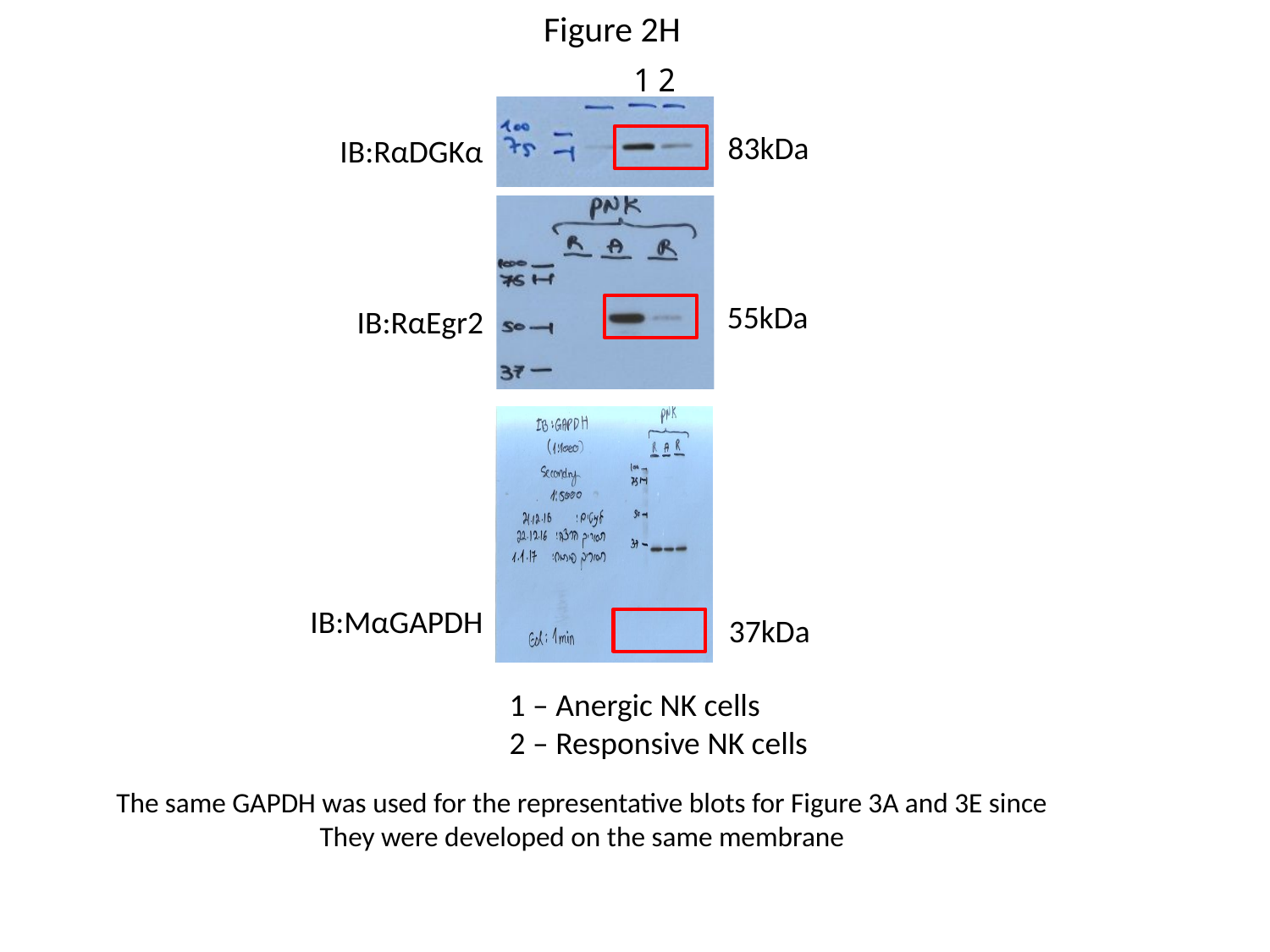

# Figure 2H
1
2
83kDa
IB:RαDGKα
55kDa
IB:RαEgr2
IB:MαGAPDH
37kDa
1 – Anergic NK cells
2 – Responsive NK cells
The same GAPDH was used for the representative blots for Figure 3A and 3E since
They were developed on the same membrane
