# Supplementary figures and images for: Dysfunctional natural killer cells can be reprogrammed to regain anti-tumor activity
Source: EMBO J. 2024 Apr 18;43(13):2552–81. doi: 10.1038/s44318-024-00094-5 (PMC11217363; doi:10.1038/s44318-024-00094-5)

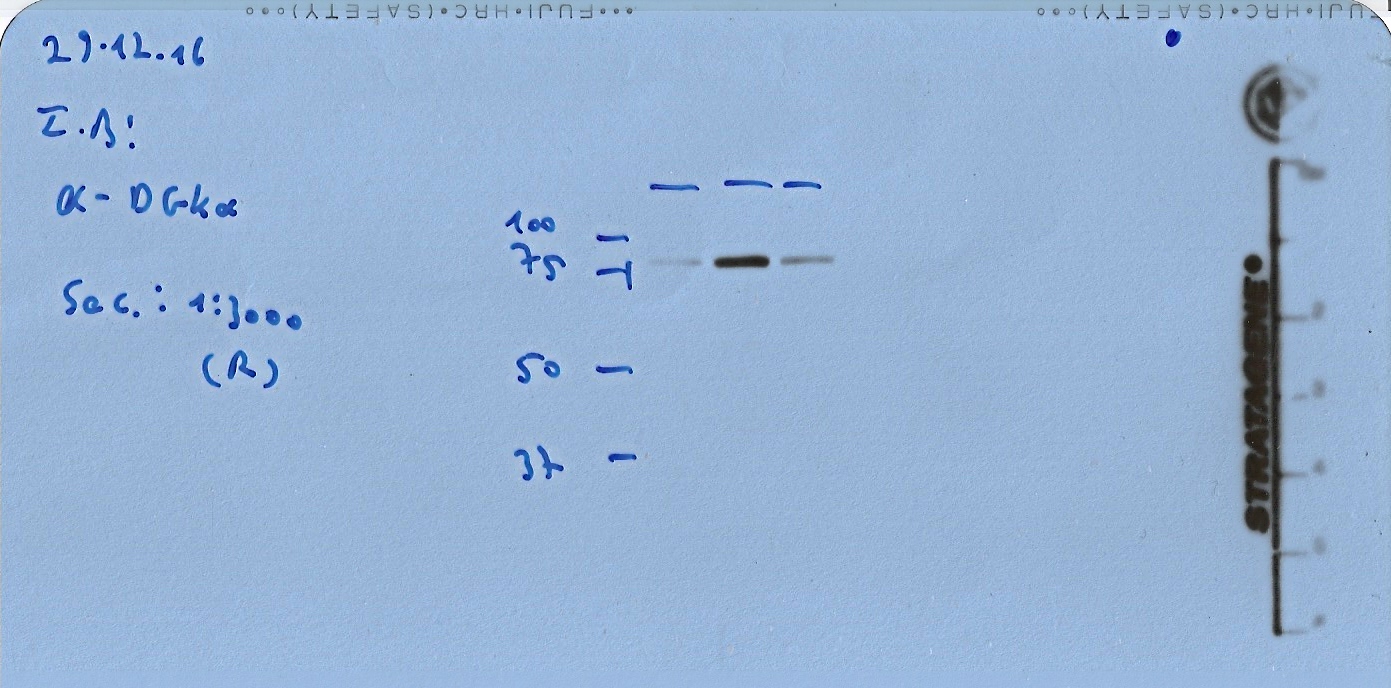

Supplement: Supplementary file 3 — Source data Fig. 2 [file 44318_2024_94_MOESM3_ESM.zip › Figure 2/Blot uncut images/DGKa.jpg]

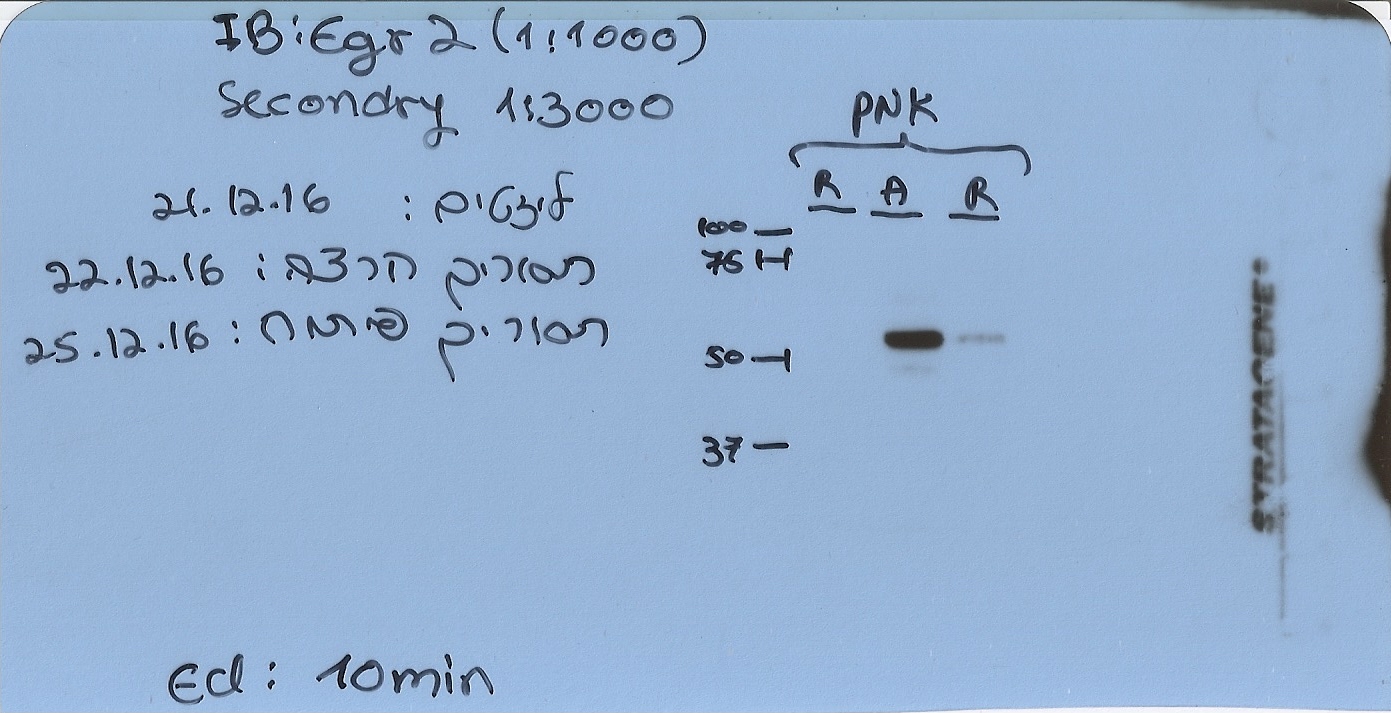

Supplement: Supplementary file 3 — Source data Fig. 2 [file 44318_2024_94_MOESM3_ESM.zip › Figure 2/Blot uncut images/Egr2 10 min.jpg]

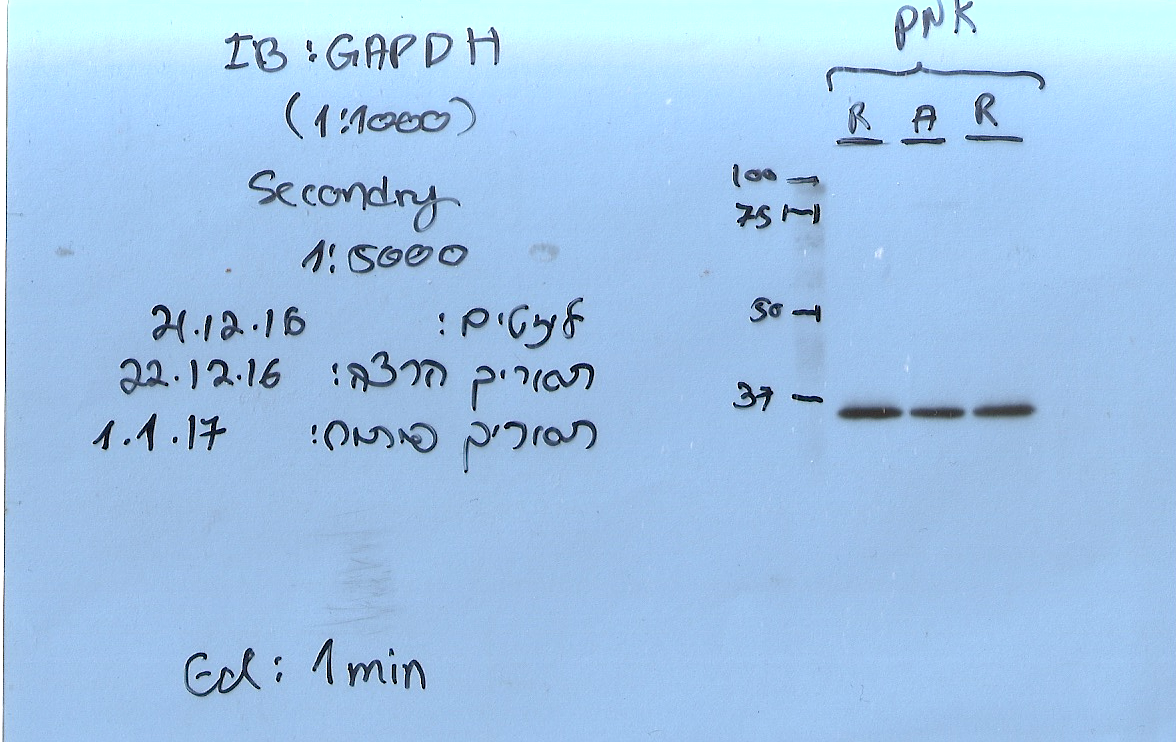

Supplement: Supplementary file 3 — Source data Fig. 2 [file 44318_2024_94_MOESM3_ESM.zip › Figure 2/Blot uncut images/GAPDH 1 min 21.12.16.tif]

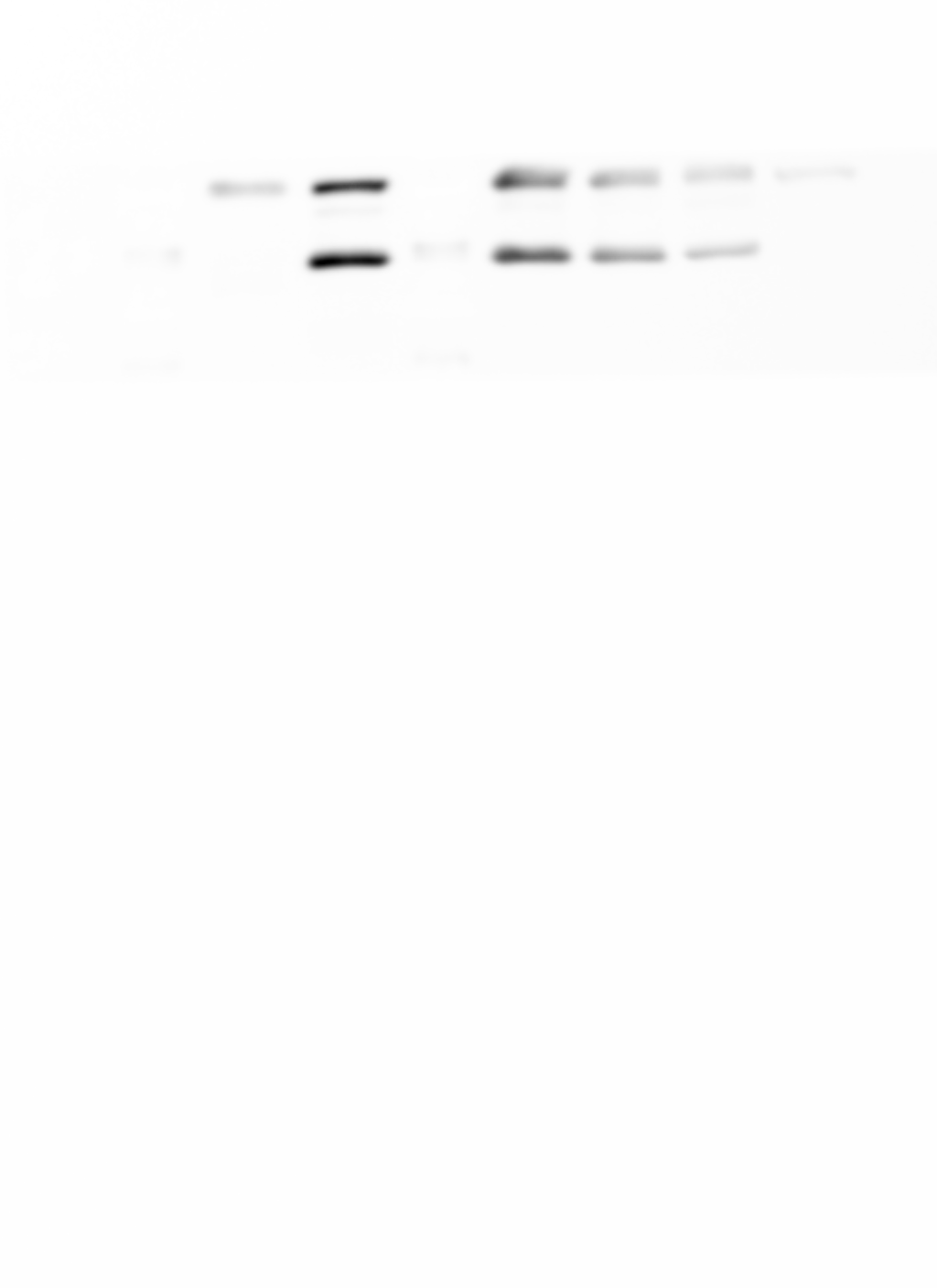

Supplement: Supplementary file 5 — Source data Fig. 4 [file 44318_2024_94_MOESM5_ESM.zip › Figure 4/Uncut Blot/pNK A&R DGKa (o) EGR2 10s 2021.08.24_17.14.30_Ch/24 Aug 21_pNK A&R DGKa 10s 2021.08.24_17.14.30_Ch.tif]

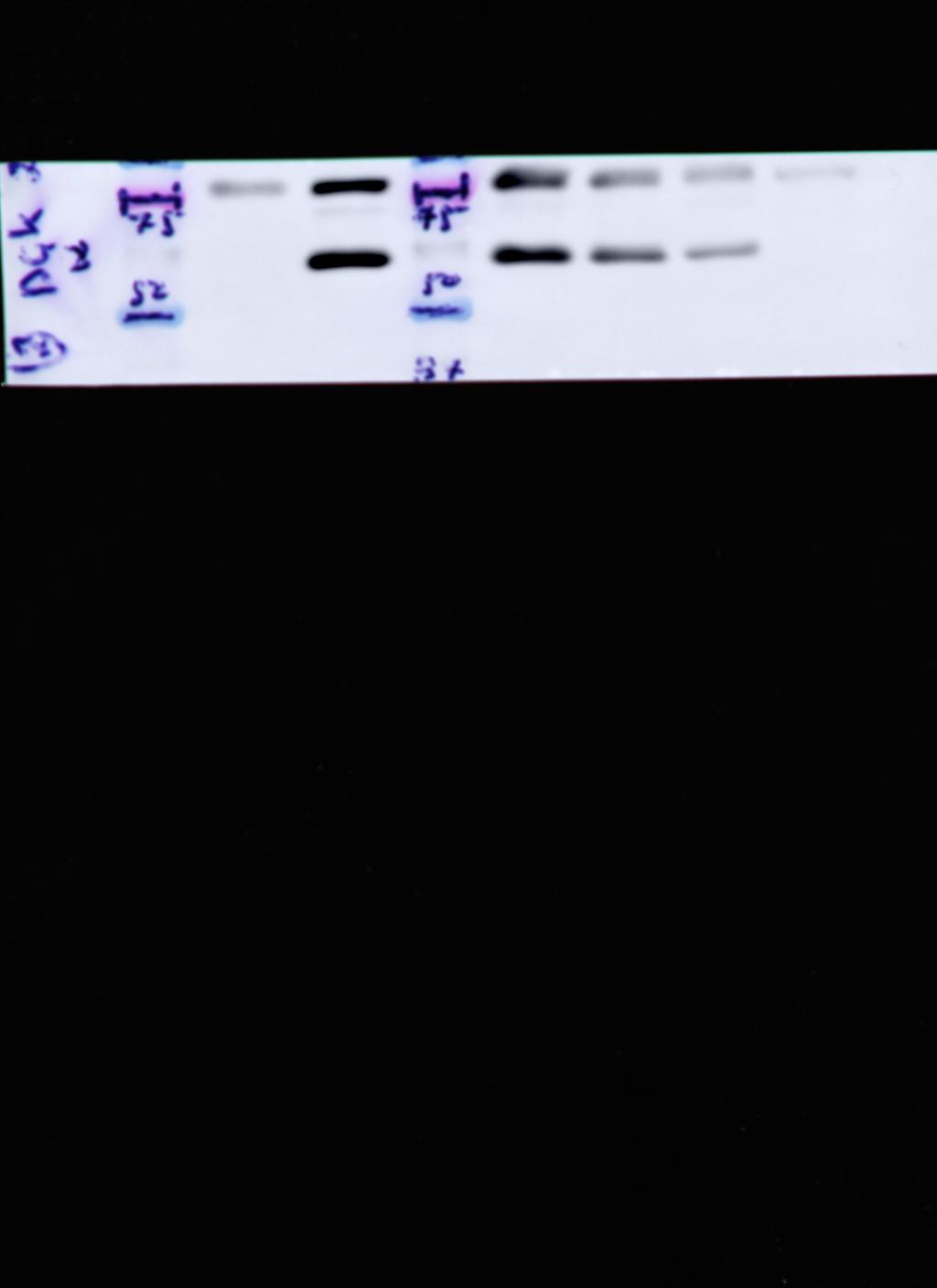

Supplement: Supplementary file 5 — Source data Fig. 4 [file 44318_2024_94_MOESM5_ESM.zip › Figure 4/Uncut Blot/pNK A&R DGKa (o) EGR2 10s 2021.08.24_17.14.30_Ch/24 Aug 21_pNK A&R DGKa 10s 2021.08.24_17.14.30_Ch+Marker.jpg]

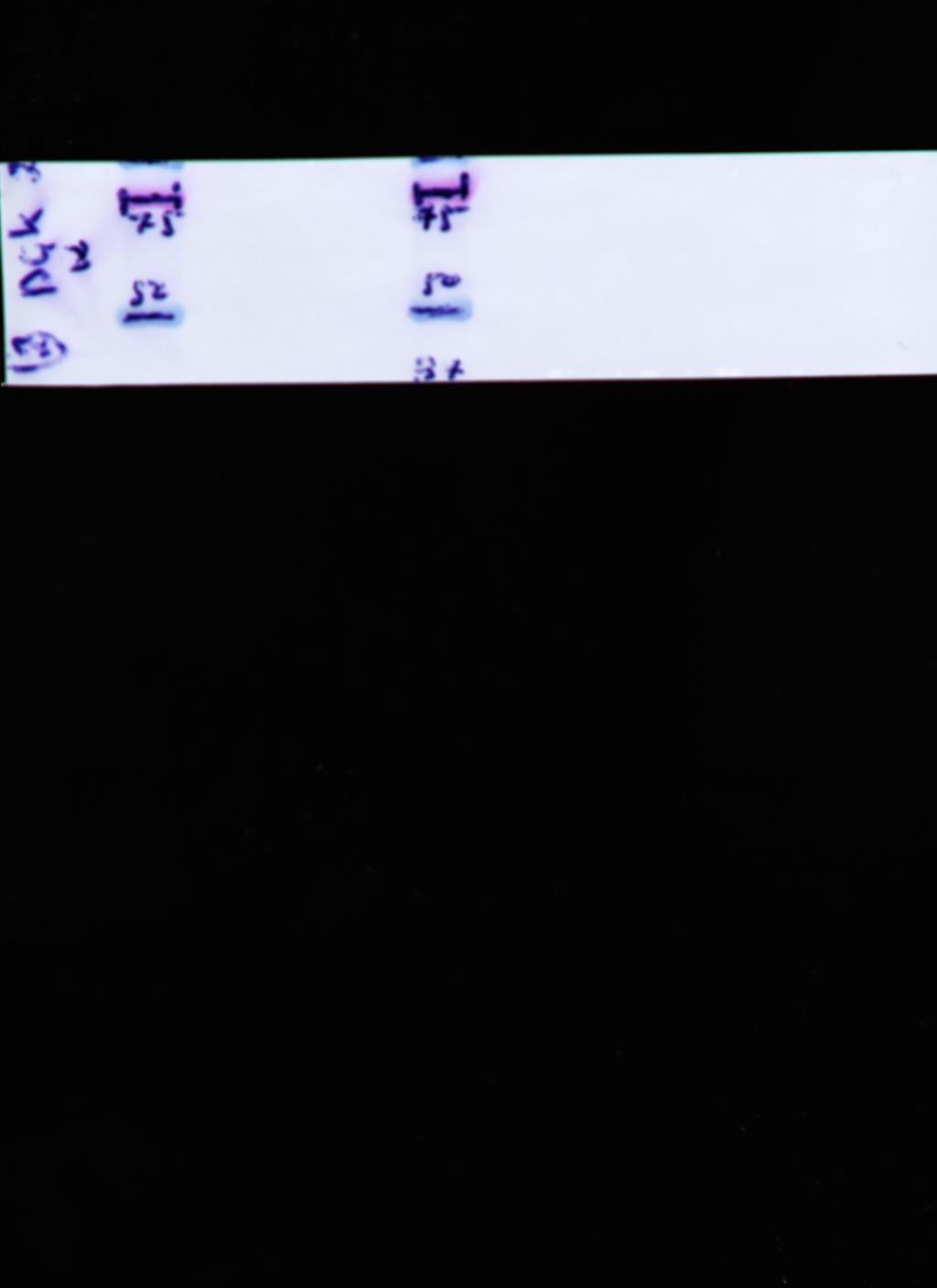

Supplement: Supplementary file 5 — Source data Fig. 4 [file 44318_2024_94_MOESM5_ESM.zip › Figure 4/Uncut Blot/pNK A&R DGKa (o) EGR2 10s 2021.08.24_17.14.30_Ch/24 Aug 21_pNK A&R DGKa 10s 2021.08.24_17.14.30_Ch-Marker.jpg]

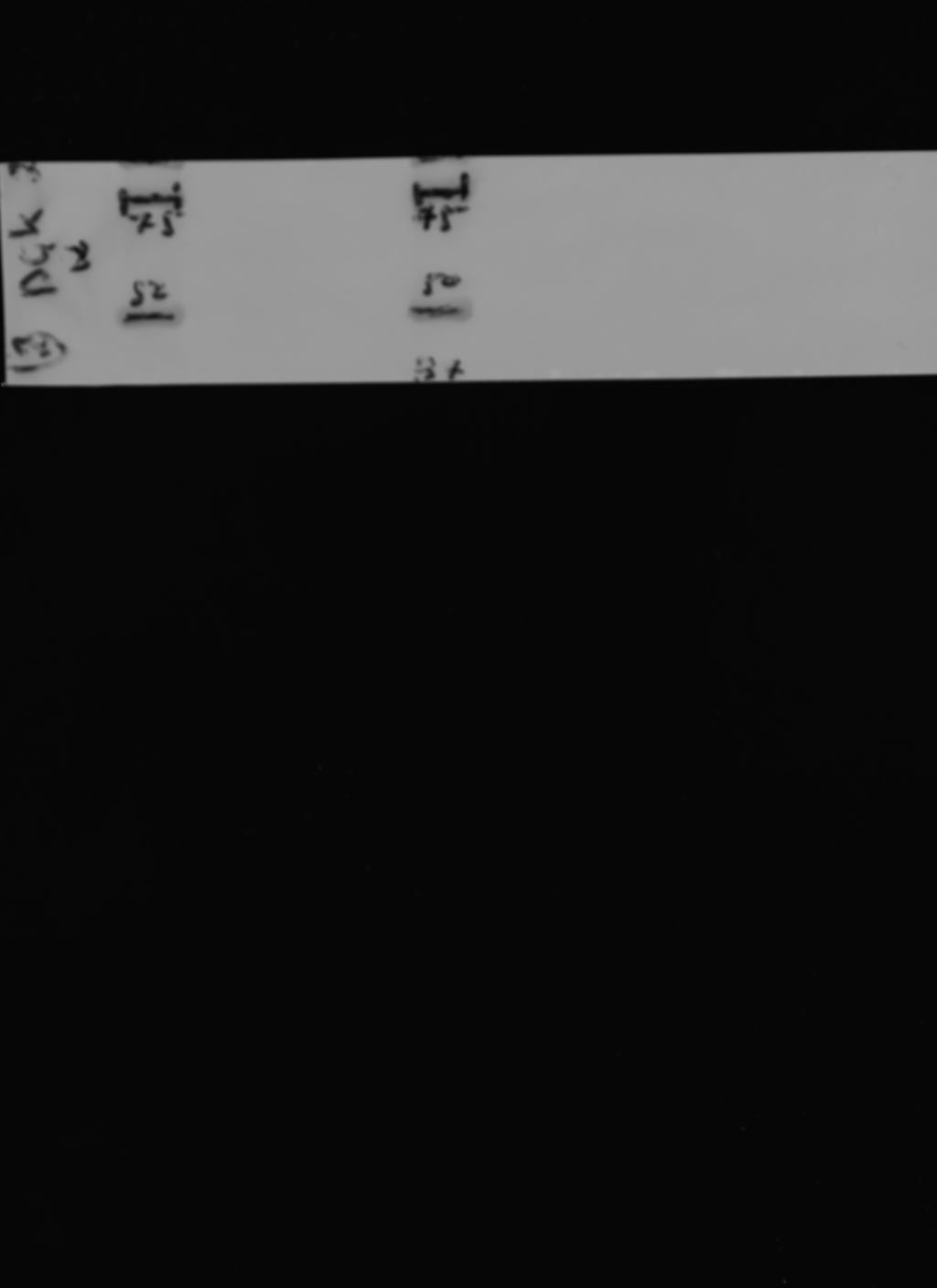

Supplement: Supplementary file 5 — Source data Fig. 4 [file 44318_2024_94_MOESM5_ESM.zip › Figure 4/Uncut Blot/pNK A&R DGKa (o) EGR2 10s 2021.08.24_17.14.30_Ch/24 Aug 21_pNK A&R DGKa 10s 2021.08.24_17.14.30_Ch-Marker.tif]

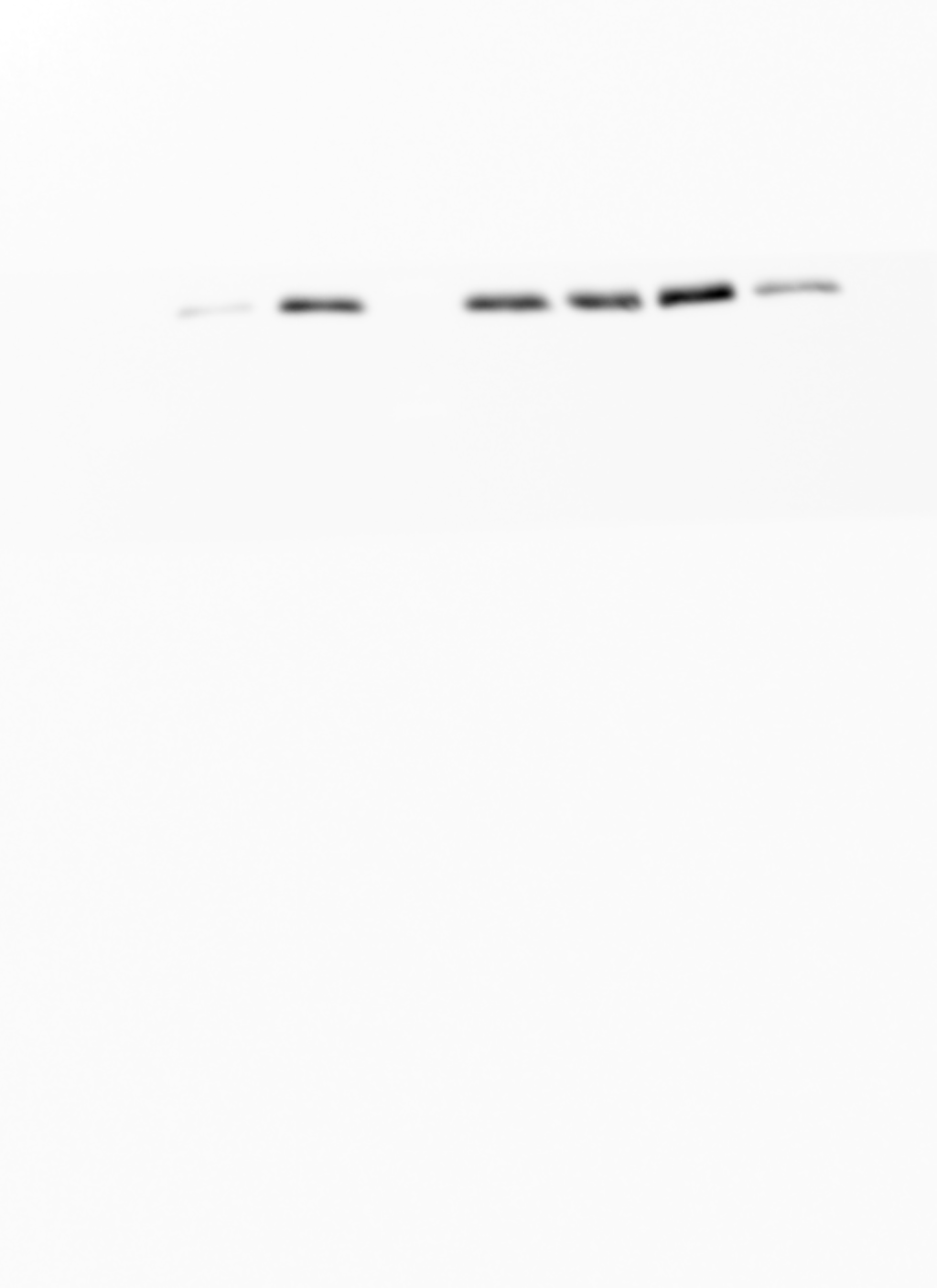

Supplement: Supplementary file 5 — Source data Fig. 4 [file 44318_2024_94_MOESM5_ESM.zip › Figure 4/Uncut Blot/pNK A&R GAPDH 5s 2021.08.24_16.42.09_Ch/24 Aug 21_pNK A&R GAPDH 5s 2021.08.24_16.42.09_Ch.tif]

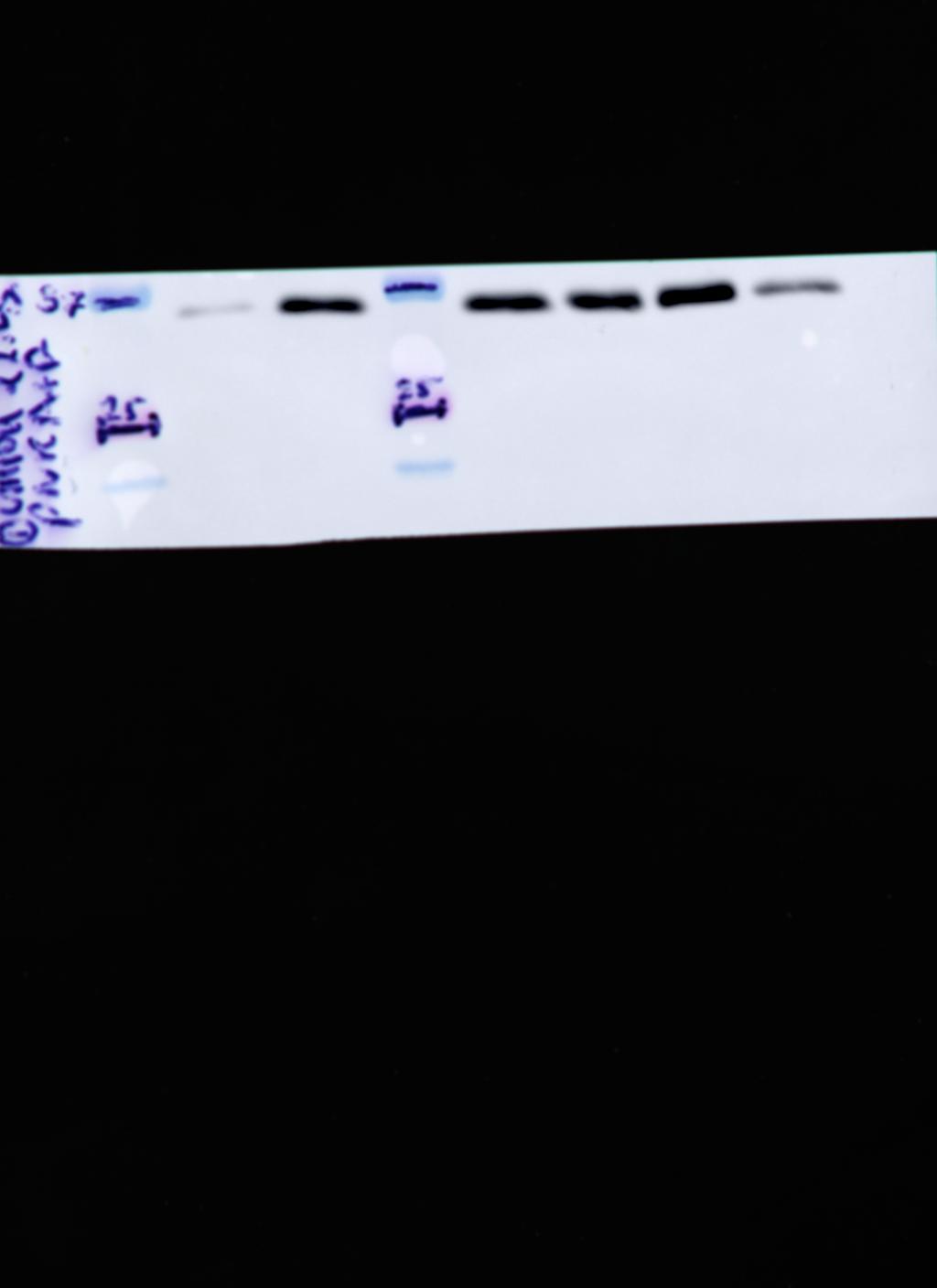

Supplement: Supplementary file 5 — Source data Fig. 4 [file 44318_2024_94_MOESM5_ESM.zip › Figure 4/Uncut Blot/pNK A&R GAPDH 5s 2021.08.24_16.42.09_Ch/24 Aug 21_pNK A&R GAPDH 5s 2021.08.24_16.42.09_Ch+Marker.jpg]

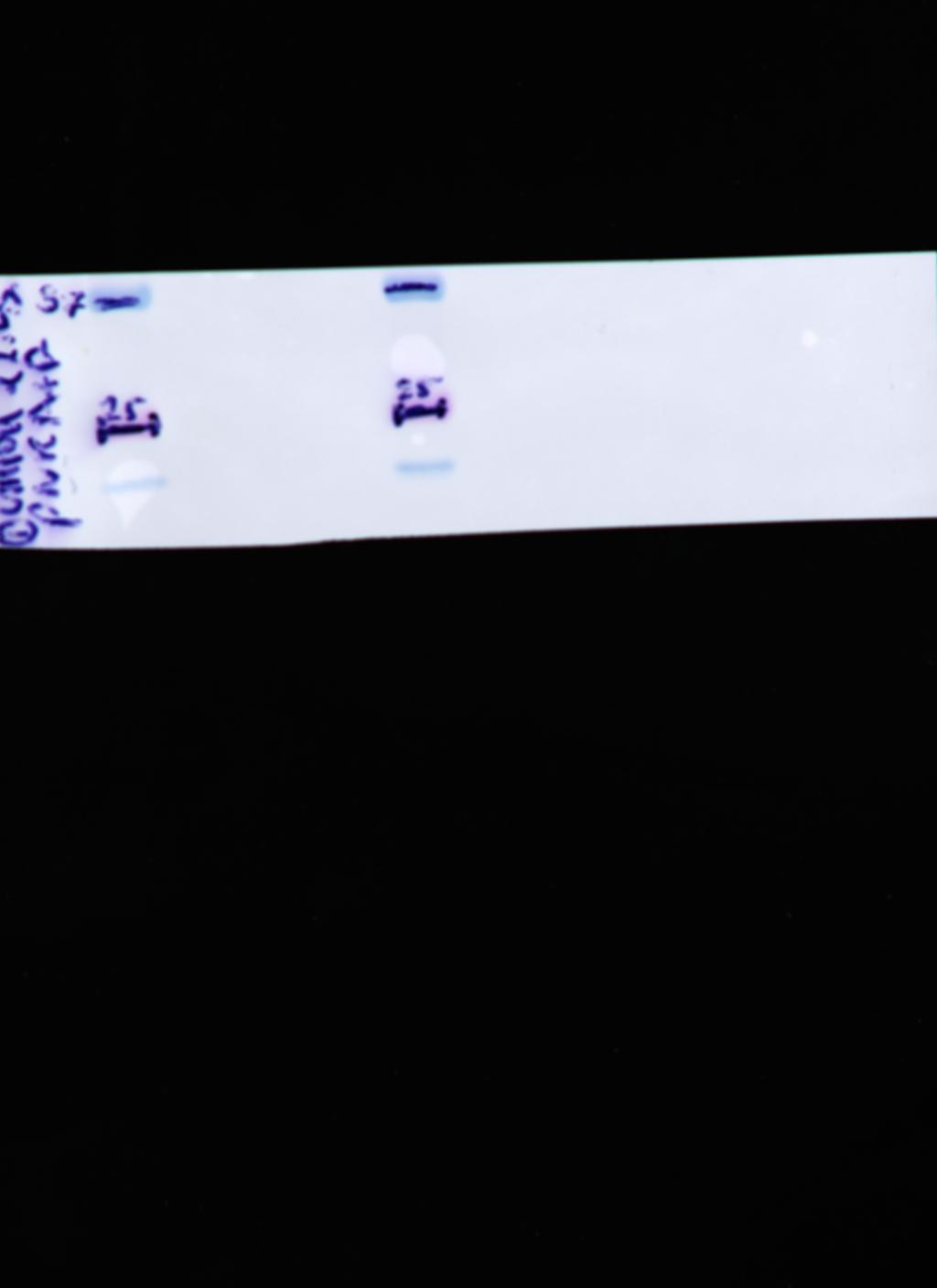

Supplement: Supplementary file 5 — Source data Fig. 4 [file 44318_2024_94_MOESM5_ESM.zip › Figure 4/Uncut Blot/pNK A&R GAPDH 5s 2021.08.24_16.42.09_Ch/24 Aug 21_pNK A&R GAPDH 5s 2021.08.24_16.42.09_Ch-Marker.jpg]

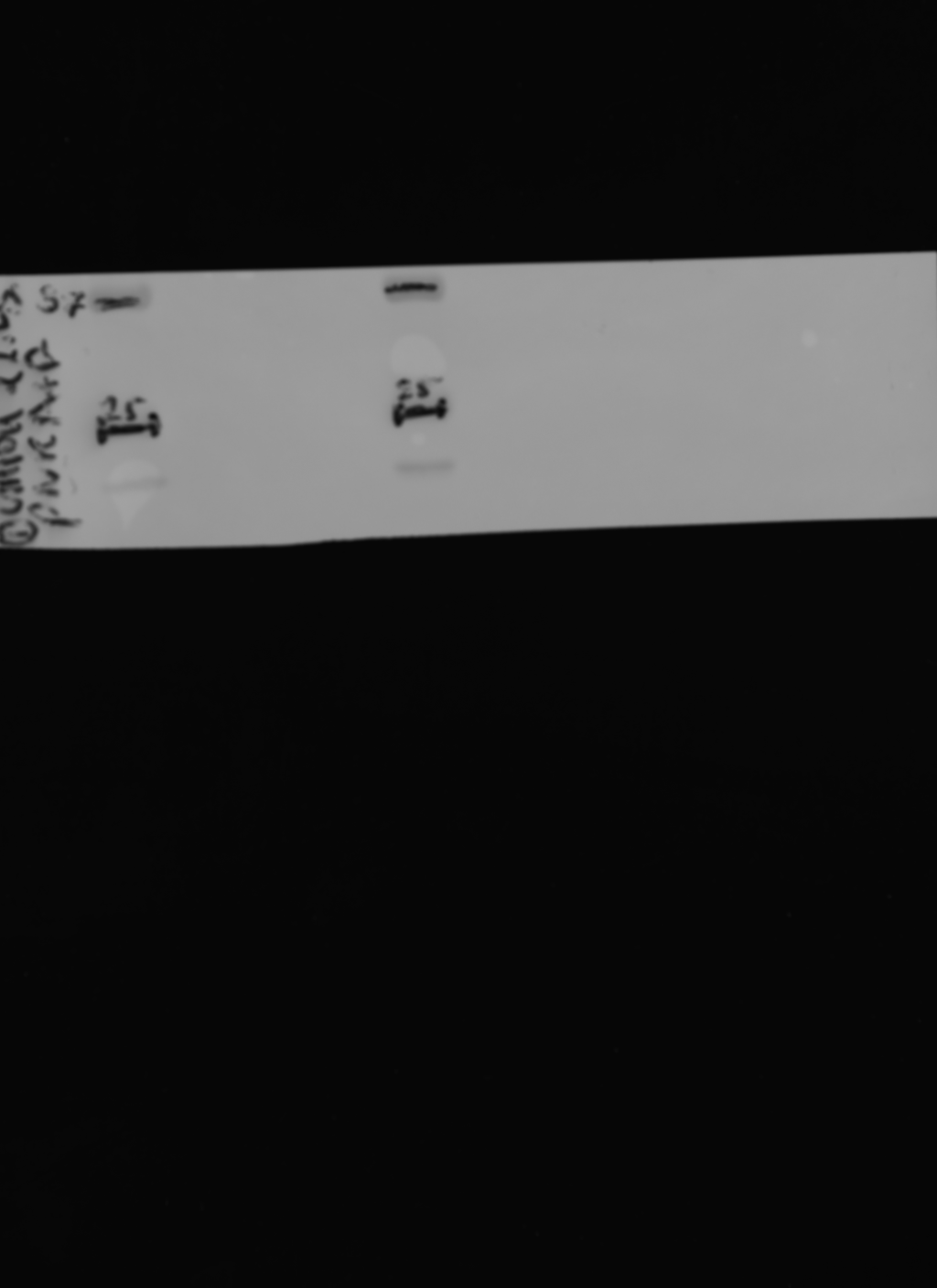

Supplement: Supplementary file 5 — Source data Fig. 4 [file 44318_2024_94_MOESM5_ESM.zip › Figure 4/Uncut Blot/pNK A&R GAPDH 5s 2021.08.24_16.42.09_Ch/24 Aug 21_pNK A&R GAPDH 5s 2021.08.24_16.42.09_Ch-Marker.tif]
