# Supplementary material for: Dysfunctional natural killer cells can be reprogrammed to regain anti-tumor activity
Source: EMBO J. 2024 Apr 18;43(13):2552–81. doi: 10.1038/s44318-024-00094-5 (PMC11217363; doi:10.1038/s44318-024-00094-5)
Supplement: Supplementary file 5 — Source data Fig. 4 [file 44318_2024_94_MOESM5_ESM.zip › Figure 4/Representative blot - Fig. 4D.pptx]

## Slide 1
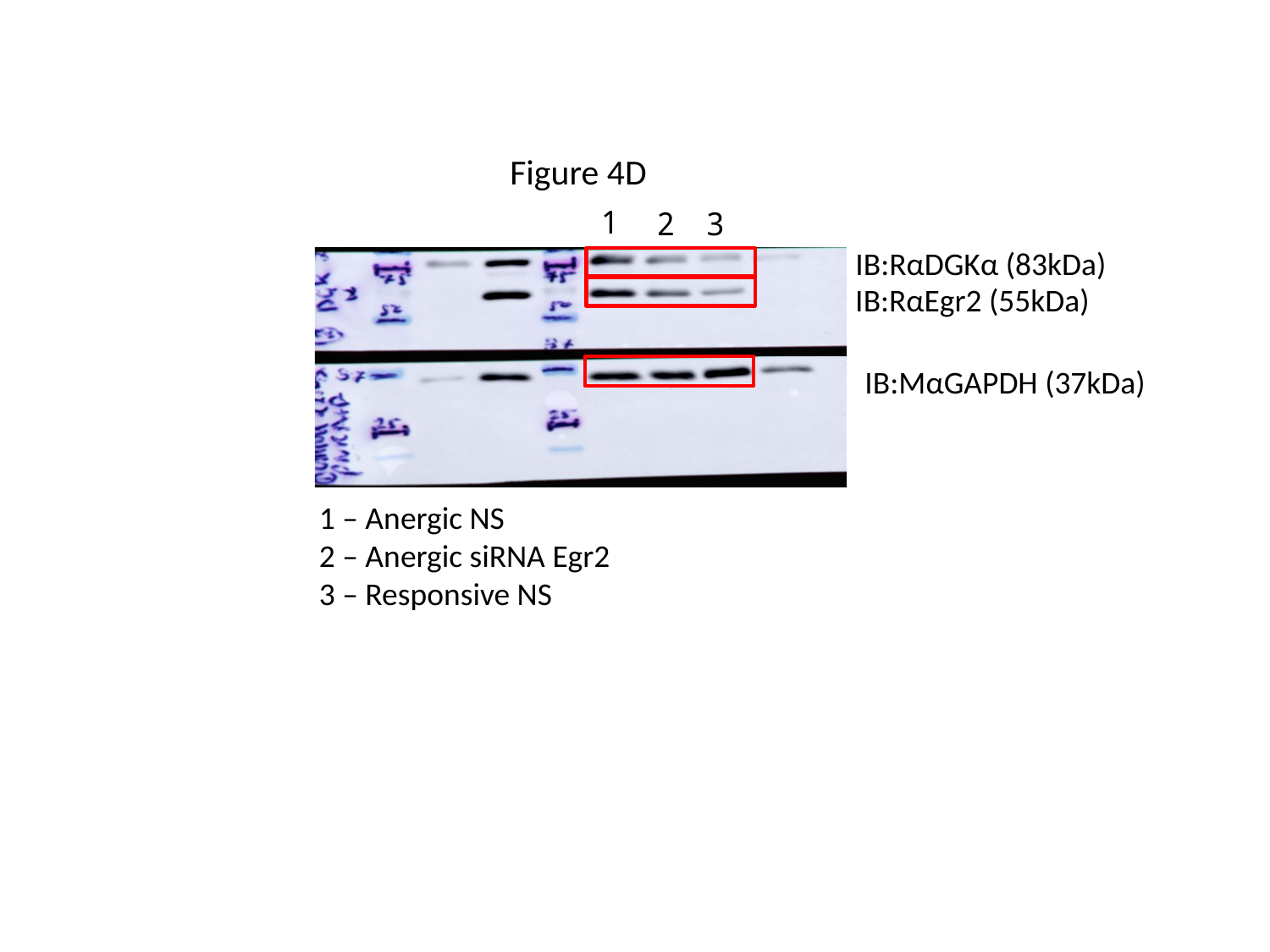

Figure 4D
1
2
3
IB:RαDGKα (83kDa)
IB:RαEgr2 (55kDa)
IB:MαGAPDH (37kDa)
1 – Anergic NS
2 – Anergic siRNA Egr2
3 – Responsive NS
