# Supplementary material for: Dysfunctional natural killer cells can be reprogrammed to regain anti-tumor activity
Source: EMBO J. 2024 Apr 18;43(13):2552–81. doi: 10.1038/s44318-024-00094-5 (PMC11217363; doi:10.1038/s44318-024-00094-5)
Supplement: Supplementary file 9 — Expanded View Figures [file 44318_2024_94_MOESM9_ESM.pdf]

Expanded View Figures

Figure EV1. RNA seq analysis—gene and pathway enrichment.

(**A**) Graphical presentation of NK isolation process -gating strategy. Primary human NK cells were isolated from PBMCs using negative selection; following purity check by $CD3^{-}CD56^{+}$ expression, the cells were stained for NKG2A PE and panKIR PE and further sorted to anergic and responsive NK cell subsets based on PE expression ($NKG2A^{-}$ $panKIR^{-}$ (PE negative) anergic subset; $NKG2A^{+}$ $panKIR^{+}$ (PE positive) responsive subset). (**B**, **C**) Purified anergic and responsive cells were subjected to incubation with 721.221 No HLA cell lines (E:T - 1:3) for 5 h at 37 °C, and were subjected to degranulation assay ($n = 3$ healthy donors). *P* values were calculated using two-tailed paired *t* test and are represented within the graph presented as means ± SEM. (**B**) For the S^{35} assay the target cells were labeled with S^{35}, and tumor lysis was measured ($n = 3$, where n is the number of healthy donors used to obtain the pNK cells). *P* values were calculated using a two-tailed paired *t* test and are represented within the graph presented as means ± SEM. (**C**) Metascape analysis of key genes and the associated enriched pathways from KEGG and GO, upregulated in the (**D**) responsive, and (**E**) anergic populations ($n = 4$, where *n* is the number of healthy donors used to obtain the pNK cells). (**F**, **G**) Metascape analysis of key genes and the enriched terms annotated to pathways from various datasets associated with the top-hit significant genes (**F**), and the TFs reflecting the top-significant genes upregulated in the anergic subset, obtained via enrichment analysis in TRRUST (**G**). *P* values for (**D**–**G**) were obtained using Hypergeometric test and Fisher's exact test. (**H**) The interactome cluster of the enriched terms annotated to pathways obtained from the significant DEGs. Source data are available online for this figure.

© The Author(s)

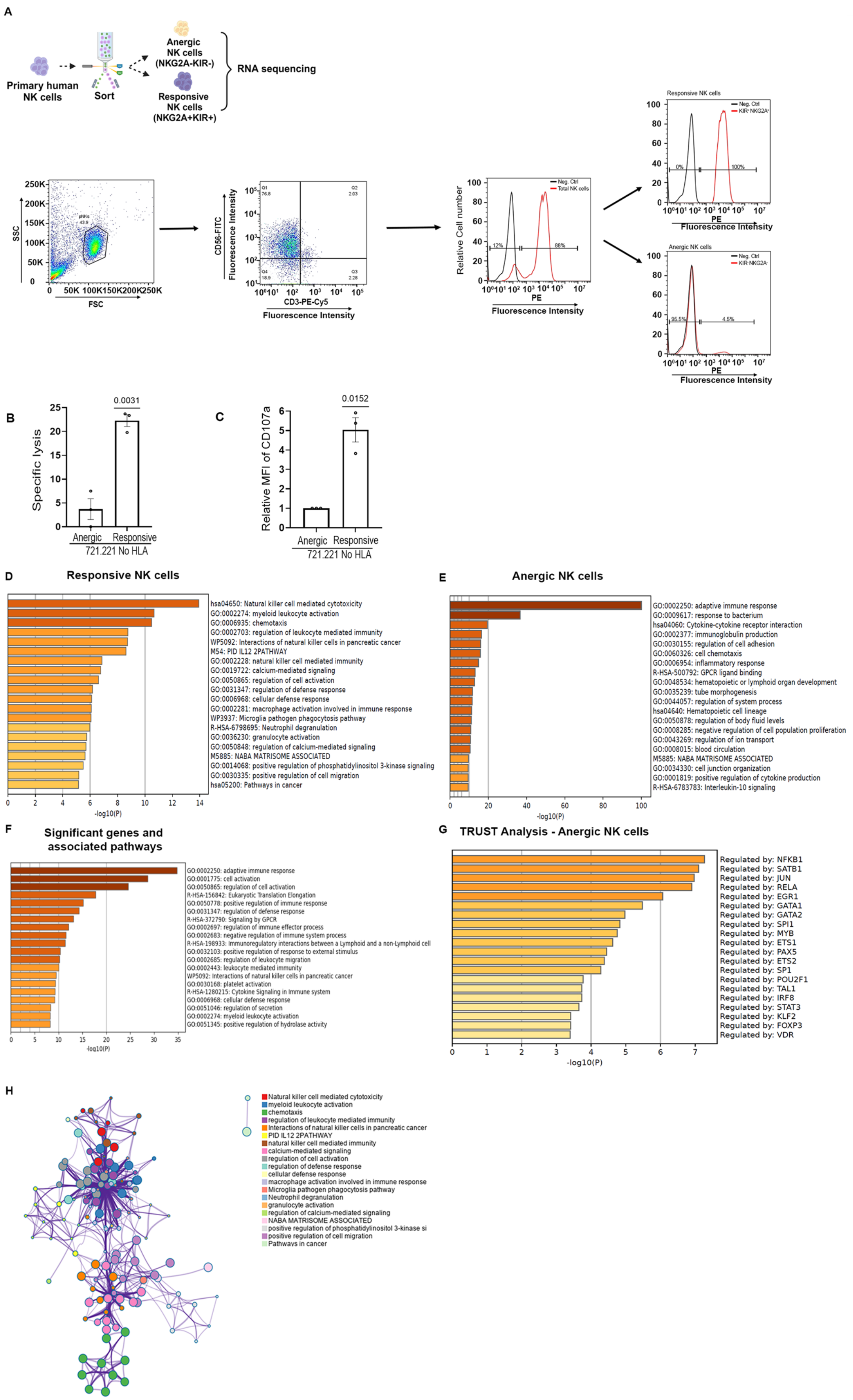
A
Primary human NK cells
Sort
Anergic NK cells (NKG2A-KIR-)
Responsive NK cells (NKG2A+KIR+)
RNA sequencing
SSC
FSC
CD56-FITC Fluorescence Intensity
CD3-PE-Cy5 Fluorescence Intensity
Relative Cell number
PE Fluorescence Intensity
Neg. Ctrl
Total NK cells
12%
88%
Responsive NK cells
0%
100%
Anergic NK cells
95.5%
4.5%
B
Specific lysis
0.0031
Anergic
Responsive
721.221 No HLA
C
Relative MFI of CD107a
0.0152
Anergic
Responsive
721.221 No HLA
D
Responsive NK cells
hsa04650: Natural killer cell mediated cytotoxicity
GO:0002274: myeloid leukocyte activation
GO:0006935: chemotaxis
GO:0002703: regulation of leukocyte mediated immunity
WP5092: Interactions of natural killer cells in pancreatic cancer
M54: PID IL12 2PATHWAY
GO:0002228: natural killer cell mediated immunity
GO:0019722: calcium-mediated signaling
GO:0050865: regulation of cell activation
GO:0031347: regulation of defense response
GO:0006968: cellular defense response
GO:0002281: macrophage activation involved in immune response
WP3937: Microglia pathogen phagocytosis pathway
R-HSA-6798695: Neutrophil degranulation
GO:0036230: granulocyte activation
GO:0050848: regulation of calcium-mediated signaling
M5885: NABA MATRISOME ASSOCIATED
GO:0014068: positive regulation of phosphatidylinositol 3-kinase signaling
GO:0030335: positive regulation of cell migration
hsa05200: Pathways in cancer
-log10(P)
E
Anergic NK cells
GO:0002250: adaptive immune response
GO:0009617: response to bacterium
hsa04060: Cytokine-cytokine receptor interaction
GO:0002377: immunoglobulin production
GO:0030155: regulation of cell adhesion
GO:0060326: cell chemotaxis
GO:0006954: inflammatory response
R-HSA-500792: GPCR ligand binding
GO:0048534: hematopoietic or lymphoid organ development
GO:0035239: tube morphogenesis
GO:0044057: regulation of system process
hsa04640: Hematopoietic cell lineage
GO:0050878: regulation of body fluid levels
GO:0008285: negative regulation of cell population proliferation
GO:0043269: regulation of ion transport
GO:0008015: blood circulation
M5885: NABA MATRISOME ASSOCIATED
GO:0034330: cell junction organization
GO:0001819: positive regulation of cytokine production
R-HSA-6783783: Interleukin-10 signaling
-log10(P)
F
Significant genes and associated pathways
GO:0002250: adaptive immune response
GO:0001775: cell activation
GO:0050865: regulation of cell activation
R-HSA-156842: Eukaryotic Translation Elongation
GO:0050778: positive regulation of immune response
GO:0031347: regulation of defense response
R-HSA-372790: Signaling by GPCR
GO:0002697: regulation of immune effector process
GO:0002683: negative regulation of immune system process
R-HSA-198933: Immunoregulatory interactions between a Lymphoid and a non-Lymphoid cell
GO:0032103: positive regulation of response to external stimulus
GO:0002685: regulation of leukocyte migration
GO:0002443: leukocyte mediated immunity
WP5092: Interactions of natural killer cells in pancreatic cancer
GO:0030168: platelet activation
R-HSA-1280215: Cytokine Signaling in Immune system
GO:0006968: cellular defense response
GO:0051046: regulation of secretion
GO:0002274: myeloid leukocyte activation
GO:0051345: positive regulation of hydrolase activity
-log10(P)
G
TRUST Analysis - Anergic NK cells
Regulated by: NFKB1
Regulated by: SATB1
Regulated by: JUN
Regulated by: RELA
Regulated by: EGR1
Regulated by: GATA1
Regulated by: GATA2
Regulated by: SPI1
Regulated by: MYB
Regulated by: ETS1
Regulated by: PAX5
Regulated by: ETS2
Regulated by: SP1
Regulated by: POU2F1
Regulated by: TAL1
Regulated by: IRF8
Regulated by: STAT3
Regulated by: KLF2
Regulated by: FOXP3
Regulated by: VDR
-log10(P)
H
Natural killer cell mediated cytotoxicity
myeloid leukocyte activation
chemotaxis
regulation of leukocyte mediated immunity
Interactions of natural killer cells in pancreatic cancer
PID IL12 2PATHWAY
natural killer cell mediated immunity
calcium-mediated signaling
regulation of cell activation
regulation of defense response
cellular defense response
macrophage activation involved in immune response
Microglia pathogen phagocytosis pathway
Neutrophil degranulation
granulocyte activation
regulation of calcium-mediated signaling
NABA MATRISOME ASSOCIATED
positive regulation of phosphatidylinositol 3-kinase si
positive regulation of cell migration
Pathways in cancer

 © The Author(s)

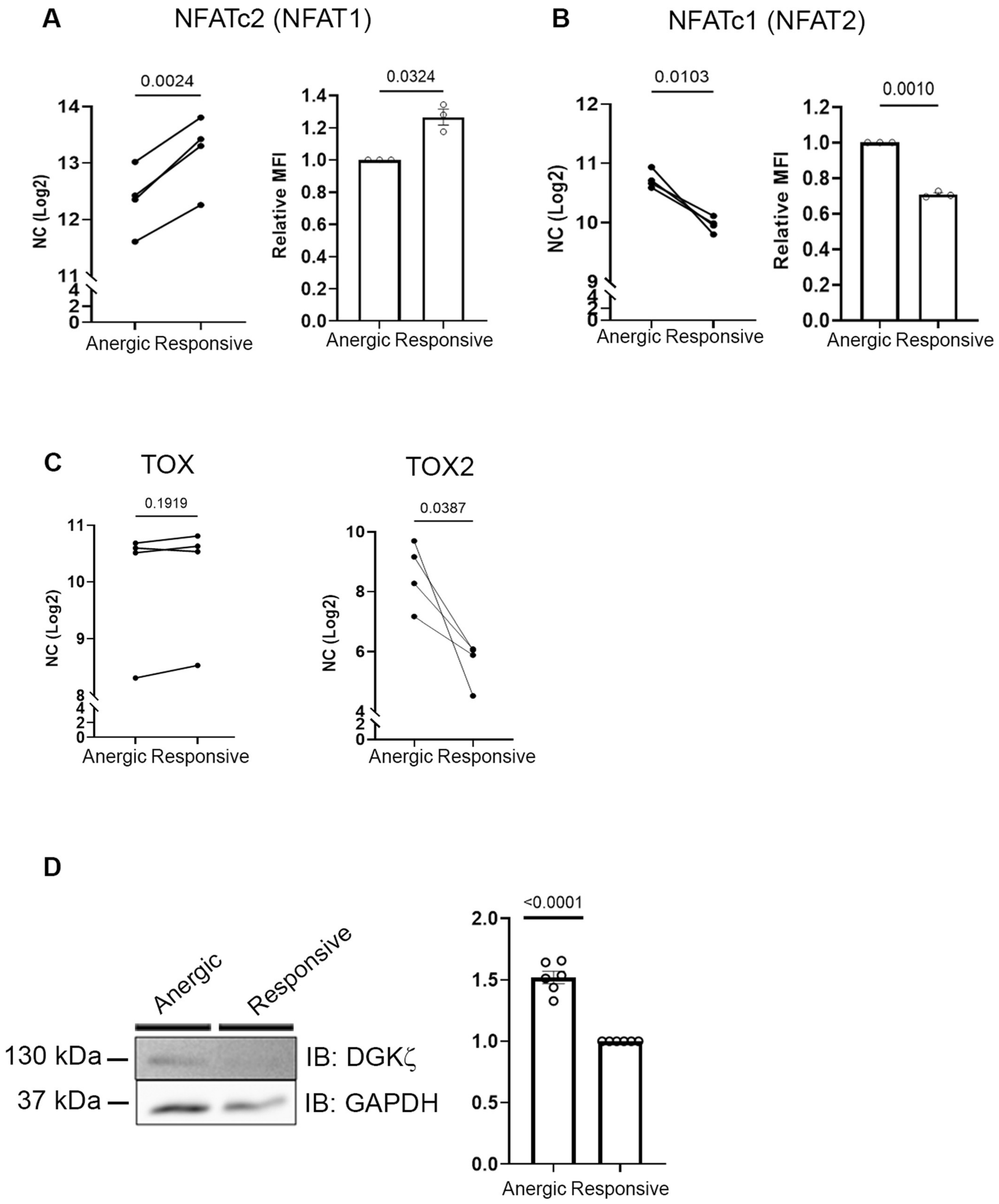
A
NFATc2 (NFAT1)
0.0024
NC (Log2)
Anergic Responsive
0.0324
Relative MFI
B
NFATc1 (NFAT2)
0.0103
0.0010
C
TOX
0.1919
TOX2
0.0387
D
Anergic
Responsive
130 kDa
37 kDa
IB: DGKζ
IB: GAPDH
<0.0001

Figure EV2. RNA seq analysis and DGKα expression levels.

(**A**, **B**) Transcript level by RNA seq (left) ($n = 4$, healthy donors used to obtain the pNK) and protein expression ($n = 3$ healthy donors) via flow cytometry (right) of (**A**) NFAT1 (NFATc2) and (**B**) NFAT2 (NFAT c1), respectively. The *P* value was calculated using a two-tailed *t* test, and the number of repeats is indicated within the graph presented as means ± SEM. (**C**) The transcript levels of TOX and TOX2 in anergic vs responsive cells ($n = 4$, where *n* is the number of healthy donors used to obtain the pNK cells). *P* values were calculated using a two-tailed *t* test with pairing and are indicated within the graph; NC normalized counts. (**D**) Purified anergic and responsive NK cells were lysed and subjected to western blot analysis with DGKα antibody ($n = 6$, where *n* is the number of healthy donors used to obtain the pNK cells). *P* values were calculated using a two-tailed *t* test with pairing, and are indicated within the graph presented as means ± SEM. Source data are available online for this figure.

© The Author(s)

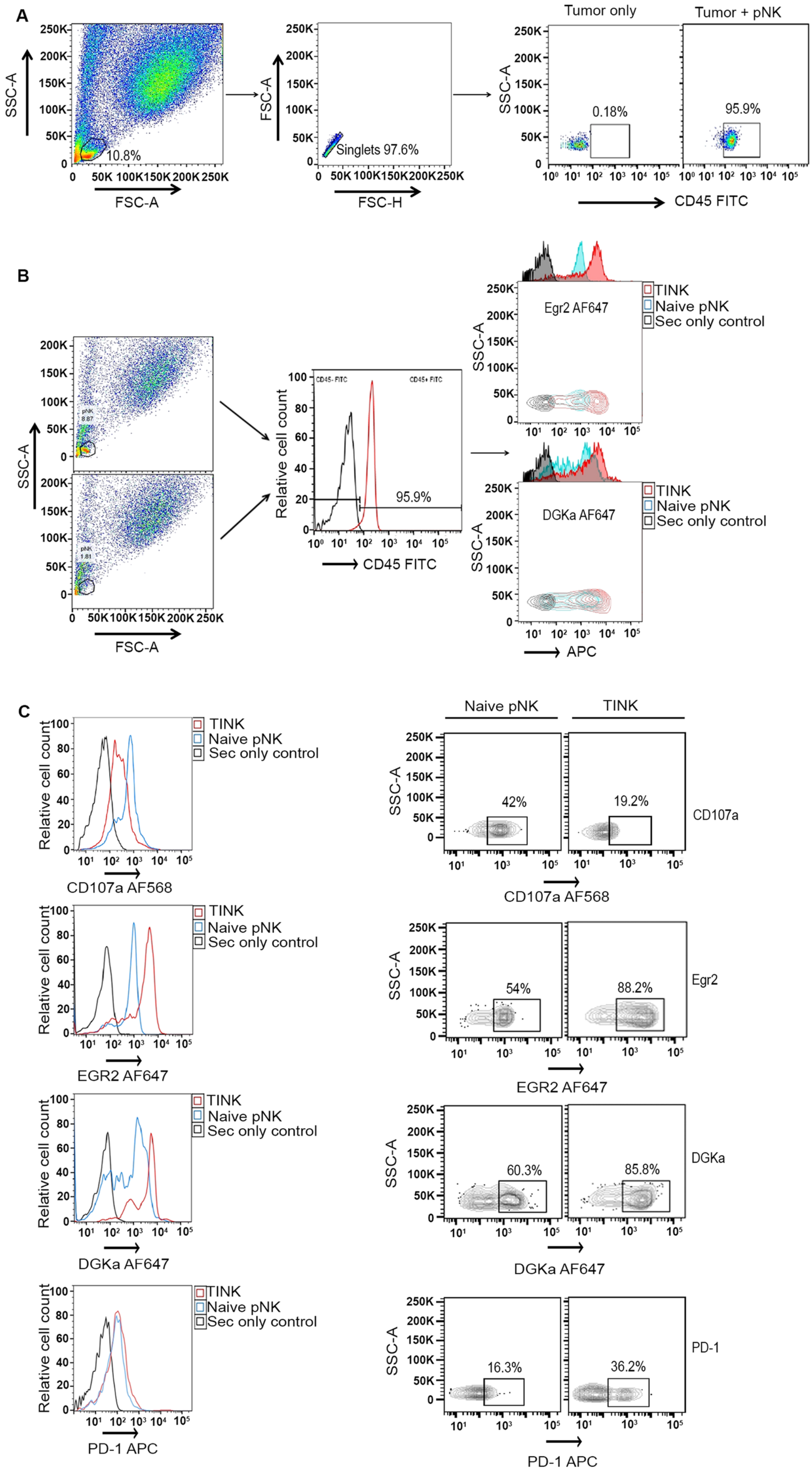
A
SSC-A
FSC-A
10.8%
FSC-A
FSC-H
Singlets 97.6%
Tumor only
Tumor + pNK
0.18%
95.9%
CD45 FITC
B
pNK
FSC-A
Relative cell count
95.9%
CD45 FITC
Egr2 AF647
DGKa AF647
TINK
Naive pNK
Sec only control
APC
C
CD107a AF568
EGR2 AF647
DGKa AF647
PD-1 APC
Naive pNK
TINK
42%
19.2%
CD107a
54%
88.2%
Egr2
60.3%
85.8%
DGKa
16.3%
36.2%
PD-1

Figure EV3. Flow cytometry gating strategy.

The gating strategy employed for the in vivo experiment. (**A**) NK cells were distinguished from the target PANC-1 cells according to FSC and SSC. CD45 (antibody specifically recognizing human (h)CD45) expression was used to further distinguish the pNK from the tumor and any other murine cells. Pseudocolor presentation is shown to identify the NK cells, which are $CD45^+$ (95.9%) referred as tumor infiltrating NK cells (TINK). (**B**) Flow cytometry analysis of intracellular staining was performed to measure pNK expression on cells from the mice with tumor-only (no NK administration) control and distinguished based on CD45 expression. The NK cells were distinguished from the target cells based on their volume and density (FSC and SSC) and were re-gated to $CD45^+$ subsets. DGKα and Egr2 expressions were measured on the $CD45^+$ gated population. Histogram offsets and MFI were used for graphical presentation. Graphs show fluorescence intensity. The *Y* axis indicates relative cell number, and the *X* axis indicates the MFI. (**C**) Representative histograms and contour plots including outliers for the data in Fig. 3C–F.

 © The Author(s)

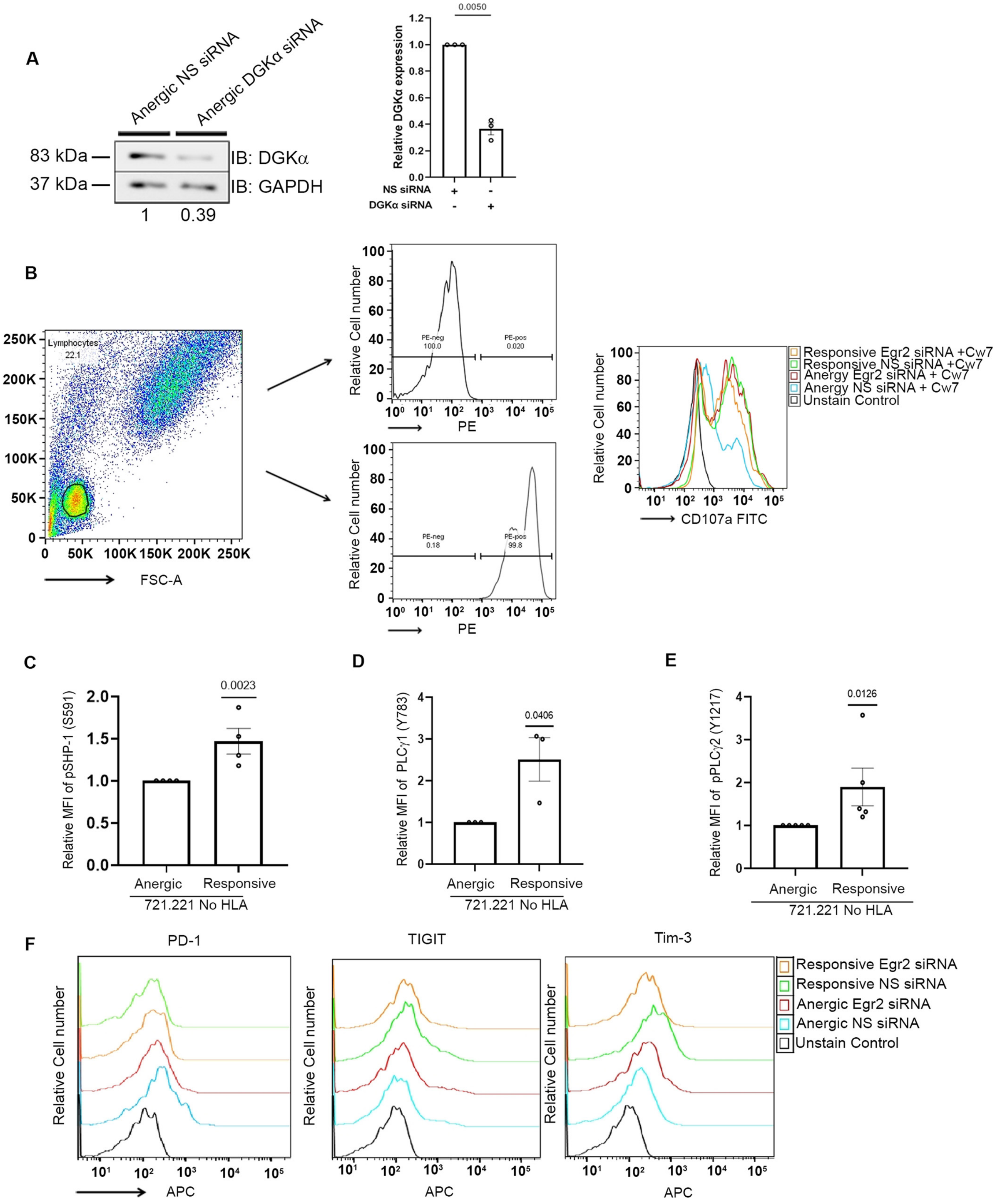
A
Anergic NS siRNA
Anergic DGKα siRNA
83 kDa
37 kDa
IB: DGKα
IB: GAPDH
1
0.39
0.0050
Relative DGKα expression
NS siRNA + -
DGKα siRNA - +
B
Lymphocytes
22.1
FSC-A
PE-neg
100.0
PE-pos
0.020
PE-neg
0.18
PE-pos
99.8
Relative Cell number
PE
Responsive Egr2 siRNA +Cw7
Responsive NS siRNA +Cw7
Anergy Egr2 siRNA + Cw7
Anergy NS siRNA + Cw7
Unstain Control
CD107a FITC
C
0.0023
Relative MFI of pSHP-1 (S591)
Anergic
Responsive
721.221 No HLA
D
0.0406
Relative MFI of PLCγ1 (Y783)
Anergic
Responsive
721.221 No HLA
E
0.0126
Relative MFI of pPLCγ2 (Y1217)
Anergic
Responsive
721.221 No HLA
F
PD-1
TIGIT
Tim-3
Relative Cell number
APC
Responsive Egr2 siRNA
Responsive NS siRNA
Anergic Egr2 siRNA
Anergic NS siRNA
Unstain Control

Figure EV4. Silencing efficiency and flow cytometry gating strategies.

(**A**) Gene silencing of DGKα. Anergic cells were treated with DGKα siRNA or NS siRNA and were then lysed and subjected to western blot analysis. One blot representative of three experiments is shown. The right panel shows a graph representing the quantified blots ($n = 3$, where n is the number of healthy donors used to obtain the pNK cells). Data are presented as mean ± SEM. *P* value was calculated using a two-tailed paired *t* test and is indicated within the graph. (**B**) Gating strategy employed for the analysis of results in Fig. 4. The NK cells and the 221 HLA-Cw7 cells were differentiated based on size and granularity (FSC-SSC) and gated for PE to distinguish the anergic (PE^{-}) versus the responsive population (PE^{+}). They were subsequently gated for CD107a, as indicated on the overlaid histograms. (**C–E**) Purified responsive and anergic NK cells were stimulated with 721.221 target cells, lysed and subjected to FACS analysis with (**C**) anti-pSHP-1(S591), (**D**) anti-pPLCγ1 (Y783), and (**E**) anti-pPLCγ2 (Y1217) antibodies. Graph summarizing the MFI of pSHP-1 ($n = 5$), pPLCγ1 ($n = 3$), and pPLCγ2 ($n = 5$) expression levels (where *n* is the number of healthy donors used to obtain the pNK cells). Data are presented as mean ± SEM. *P* value was calculated using a two-tailed *t* test with matched data repeats and is indicated within the graph. (**F**) Representative overlaid histograms for Fig. 4G–I. Source data are available online for this figure.

 © The Author(s)

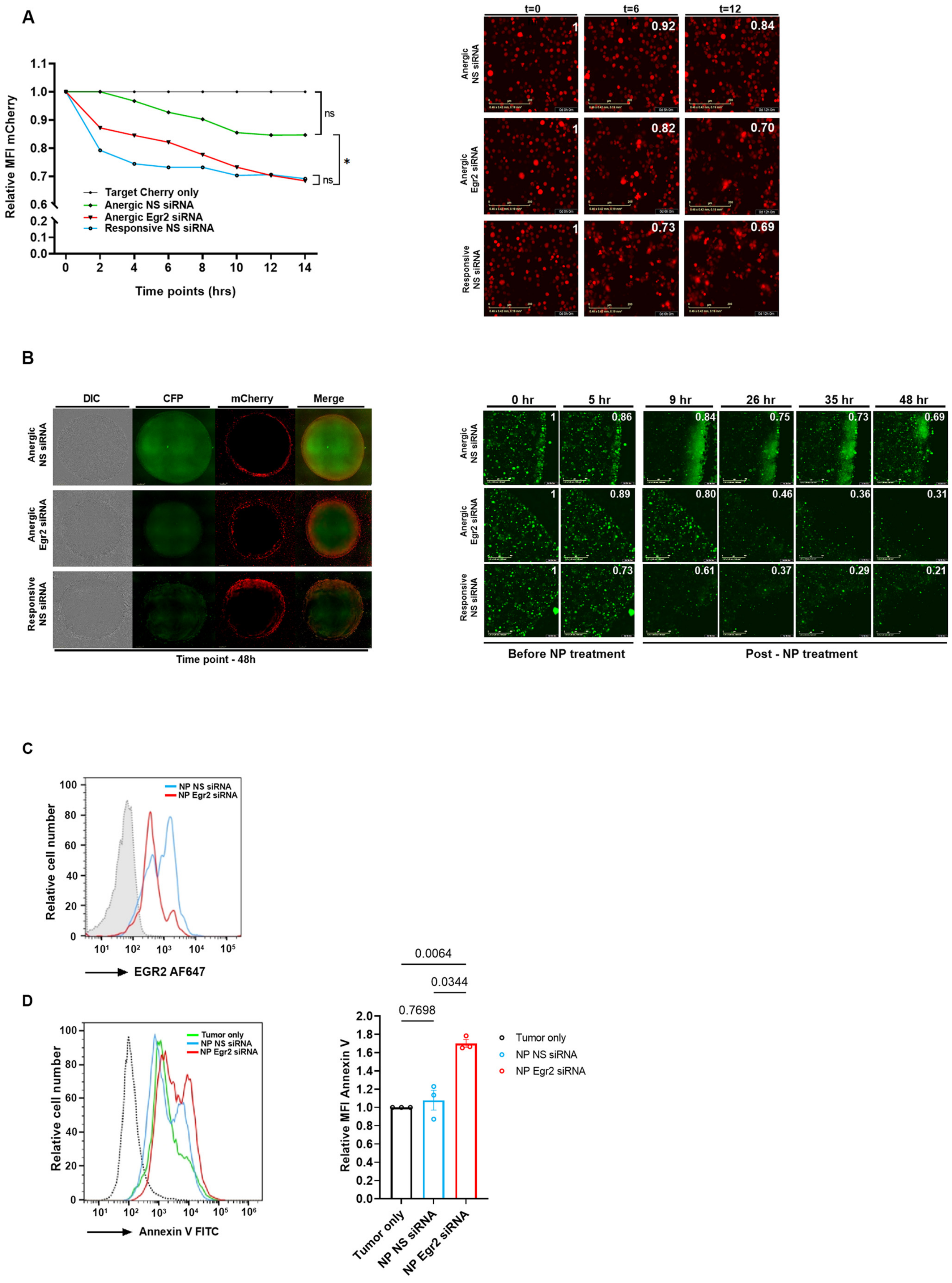
A
Relative MFI mCherry
Time points (hrs)
Target Cherry only
Anergic NS siRNA
Anergic Egr2 siRNA
Responsive NS siRNA
ns
*
ns
t=0
t=6
t=12
Anergic NS siRNA
Anergic Egr2 siRNA
Responsive NS siRNA
1
0.92
0.84
1
0.82
0.70
1
0.73
0.69
B
DIC
CFP
mCherry
Merge
Anergic NS siRNA
Anergic Egr2 siRNA
Responsive NS siRNA
Time point - 48h
0 hr
5 hr
9 hr
26 hr
35 hr
48 hr
1
0.86
0.84
0.75
0.73
0.69
1
0.89
0.80
0.46
0.36
0.31
1
0.73
0.61
0.37
0.29
0.21
Before NP treatment
Post - NP treatment
C
Relative cell number
NP NS siRNA
NP Egr2 siRNA
EGR2 AF647
D
Relative cell number
Tumor only
NP NS siRNA
NP Egr2 siRNA
Annexin V FITC
0.0064
0.0344
0.7698
Relative MFI Annexin V
Tumor only
NP NS siRNA
NP Egr2 siRNA

© The Author(s)

Figure EV5. Incucyte analysis with representative images and ex vivo analysis.

(**A**) Anergic and responsive cells were treated with either Egr2 or NS siRNA, and Incucyte-based tumor lysis assay was performed on 721.221 HLA-Cw7 target cells expressing mCherry. *P* value was cumulatively calculated for every time point using one-way ANOVA, and Tukeys' post hoc test was used for multiple comparisons, as indicated in the graph ($^{*}P < 0.05$). The analysis was performed for nine individual fields per image (16 images) (with ~$N = 50$ cells in each field). Green line: anergic NS siRNA; red line: anergic Egr2 siRNA; blue line: responsive NS siRNA. Anergic and responsive cells were transfected with EGR2 siRNA or NS siRNA, seeded with 721.221 HLA-Cw7 expressing mCherry cells at an E:T ratio of 10:1, and subjected to Incucyte imaging and analysis; the decrease in fluorescence intensity was measured, and images from the indicated time points are shown. The values were normalized to 721.221 Cherry only—no effector control. Right panel: Images showing cell fluorescence at different time points (scale bar: 200 µm). (**B**) Human CML OTS prepared using K562 CFP cells in Matrigel (1:1–v/v%), were seeded with anergic or responsive pNK cells at an E:T ratio of 5:1. After 6 h, NPs encapsulating Egr2 siRNA or NS siRNA were added to the OTS, and the decrease in fluorescence intensity was monitored and measured; the images along with the fluorescence intensity at the respective time points are shown (numbers on top right of each frame). The decrease in fluorescence over time shows the enhanced cytotoxic activity of anergic cells following Egr2 siRNA treatment (left panel: middle) similar to the responsive cells treated with NS siRNA (left panel: bottom). Whole well images of the OTS at 48 h are presented. Right panel: Images showing cell fluorescence at different time points (Scale bar: 200 µm). (**C**) The tumors were excised on day 27, a single-cell suspension was made, and cells were stained for intracellular Egr2. The pNK cells were distinguished based on FSC vs SSC, and PE content, reflecting nanoparticle incorporation. Representative histogram showing Egr2 expression levels. Blue line shows the EGR2 levels in the group treated with control NP (NS siRNA), and the red line shows the group receiving Egr2 siRNA-NP. (**D**) The dissociated tumors were then subjected to Annexin V staining for apoptotic cells. Representative histogram on the left panel shows the apoptosis of the tumors from each group (one mouse representative of three independent repeats). The right panel shows a graph summarizing the tumors obtained from three different mice. Data are presented as mean ± SEM. *P* values were calculated using one-way ANOVA with Tukeys post hoc test after normalization of the values to the "tumor only" group, which served as the control. Source data are available online for this figure.

 © The Author(s)
